# Pre-incisional infiltration with ropivacaine plus dexamethasone palmitate emulsion for postoperative pain in patients undergoing craniotomy: study protocol for a prospective, randomized controlled trial

**DOI:** 10.1186/s13063-022-06936-z

**Published:** 2022-12-12

**Authors:** Wei Zhang, Chunzhao Li, Chunmei Zhao, Nan Ji, Fang Luo

**Affiliations:** 1 Department of Day Surgery, Beijing Tiantan Hospital, Capital Medical University, Beijing, 100070 People’s Republic of China; 2 Department of Neurosurgery, Beijing Tiantan Hospital, Capital Medical University, 100070 Beijing, People’s Republic of China; 3 Department of Day Surgery and Pain Management, Beijing Tiantan Hospital, Capital Medical University, 100070 Beijing, People’s Republic of China

**Keywords:** Dexamethasone palmitate emulsion, Ropivacaine, Craniotomy, Pre-incisional infiltration, Postoperative pain

## Abstract

**Background:**

Post-craniotomy pain is a common occurrence which is associated with poor outcomes. Pre-emptive scalp infiltration with dexamethasone and ropivacaine has been proven effective in previous studies but with limited clinical significance. Dexamethasone palmitate emulsion (D-PAL) is a pro-drug incorporating dexamethasone into lipid microspheres with greater anti-inflammatory activity and fewer side effects than free dexamethasone. However, its effects in post-craniotomy pain management remain unknown. This study hypothesizes that pre-emptive scalp infiltration with ropivacaine plus D-PAL emulsion can achieve superior analgesic effects to ropivacaine alone in adult patients undergoing craniotomy.

**Methods/design:**

This is a single center, randomized controlled trial enrolling 130 patients scheduled for supratentorial craniotomy, which is expected to last longer than 4 h. We compare the efficacy and safety for postoperative pain relief of ropivacaine plus D-PAL group and ropivacaine alone group following pre-emptive scalp infiltration. Primary outcome will be pain Numerical Rating Scale at 24 h postoperatively. Secondary outcomes will include further analgesia evaluations and drug-related complications within a follow-up period of 3 months.

**Discussion:**

This is the first randomized controlled trial aiming to assess the possible benefits or disadvantages of D-PAL emulsion for incisional pain in craniotomy. It may provide an alternative to optimize pain outcome for neurosurgical patients.

**Trial registration:**

ClinicalTrials.gov (NCT04488315). Registered on 19 July 2020.

**Supplementary Information:**

The online version contains supplementary material available at 10.1186/s13063-022-06936-z.

## Background

Over two-thirds of patients have been reported to suffer moderate to excruciating incisional pain during the first 48 h after neurosurgical craniotomy [1, 2]. Inadequate pain control can predispose one to elevated intracranial pressure, leading to devastating neurological complications such as cerebral hyperemia, edema, and hemorrhage. Moreover, acute severe pain can subsequently develop into chronic pain and headache affecting up to 30% of patients, which are attributed to an increased risk of peripheral and central sensitization by the sustained noxious input [3, 4]. All these provide impetus to define the ideal options for pain management to improve postoperative care for patients undergoing craniotomy.

However, there are no guidelines for post-craniotomy pain management yet. Traditionally, systemic opioids are the mainstay of treatment. However, therapeutic doses of opioids are associated with a moderate-to-high risk of postoperative nausea and vomiting (PONV). And the postoperative neurological assessments would be delayed or interfered due to sedation and miosis caused by opioids. Also, opioid-induced respiratory depression could lead to hypoxia and hypercarbia, which subsequently increase cerebral blood flow and intracranial pressure [5]. So opioids are often used sparingly for fear of adverse effects. Other systemic analgesic drugs, for example, nonsteroidal anti-inflammatory drugs (NSAIDs), gabapentin, and ketamine, appear to be effective, but none are considered adequate to alleviate pain without risks for neurological recovery [6]. Furthermore, systemic administration is usually used after the occurrence of pain when the peripheral and central sensitizations have already developed, leading to less pronounced analgesic effects [7]. Hence, it is important to explore multimodal analgesia to provide better pain control and to minimize the reliance on systemic analgesics [8].

At present, the benefits of regional anesthesia have been positively valued, which indicate more effective and stable effects than intravenous analgesia within 24 h postoperatively [9]. Wound infiltration with local anesthetics (LAs) is the simplest, safest, and most effective regional anesthesia to prevent incisional pain in craniotomy [10]. Preemptive scalp infiltration with long-acting local anesthetics such as ropivacaine has been reported to ensure a reduced perioperative consumption of opiates and a delayed need for rescue analgesic after craniotomy [11]. However, the efficiency only lasts for a relatively short period after a long perioperative duration of craniotomy which usually exceeds 4-6 h [12]. Furthermore, previous studies have provided unsatisfactory or conflicting data in attempts to prolong the block of pain conduction by adding epinephrine or using lipid microspheres or liposome formula of local anesthetics [11, 13, 14]. Thus, the induction of sufficient and prolonged analgesic efficacy after craniotomy still remains a big challenge to clinicians.

It has been reported that post-craniotomy pain originates from the damage of muscles and soft tissues at surgical site. It is caused by the release of inflammatory mediators after incisional injury, which activate the peripheral nociceptors and lead to abnormal action potential transmitted along afferent Aδ and C-fibers [15, 16]. Therefore, drugs with a powerful local anti-inflammatory property, such as glucocorticoids, might play a pivotal role in preventing or reducing postoperative pain [17, 18]. Our research group has previously demonstrated that the addition of Diprospan (a combination of betamethasone sodium phosphate and betamethasone dipropionate) to local anesthetic ropivacaine (0.5%) successfully reduces post-craniotomy pain with an 87% decrease in opioids consumption in a follow-up of 48 h [19]. However, a recent laboratory research cautioned that the mixing of ropivacaine with betamethasone sodium phosphate could produce obviously larger crystals (> 50 μm) than with dexamethasone sodium phosphate (< 10 μm) [20]. Therefore, dexamethasone is preferred for injection in consideration of effectiveness and safety. Pre-emptive scalp infiltration with the addition of dexamethasone sodium phosphate to 0.5% ropivacaine has ensured a significant decrease in opioids consumption and pain scores within postoperative 72 h, which is consistent with physiological effects of dexamethasone with a longer half-life of 36-72 h [6, 21]. However, the positive results might have limited clinical significance concerning the absolute differences compared to the control group, which have also been observed in pediatric patients [6, 22].

Dexamethasone palmitate emulsion (D-PAL emulsion) is a pro-drug incorporating dexamethasone palmitate (D-PAL) into lipid microspheres. It is gradually hydrolyzed to dexamethasone by carboxylesterase in the reticuloendothelial system and some inflammatory cells but not in human serum, therefore indicating an uptake of 8 times higher and an anti-inflammatory activity of 5-6 times greater than its corresponding amount of free dexamethasone. Furthermore, the location of D-PAL at inflammatory lesions could reduce the risks of systemic side effects [23, 24]. The efficacy of D-PAL has been verified in multiple disorders including rheumatoid arthritis, systemic lupus erythematosus, macrophage activation syndrome, arteriosclerosis, and asthma [24–27]. For pain management, intra-articular infiltration of D-PAL in combination with mepivacaine is considered to be a safe and effective method for acute lumbar facet syndrome with no side effects [28]. Epidural injection of D-PAL has relieved edema and pain more effectively when applied after intradiscal electrothermal (IDET) treatment for discogenic pain [29]. To the best of our knowledge, no studies have evaluated the addition of D-PAL to local infiltration analgesia for neurosurgical patients. Here, we hypothesize that pre-emptive scalp infiltration with ropivacaine plus D-PAL emulsion can achieve superior analgesic effects to ropivacaine alone in adult patients undergoing craniotomy, so as to provide a clinical option of pre-emptive analgesia for post-craniotomy pain.

## Study design and methods

### Study design

**Fig. 1:**
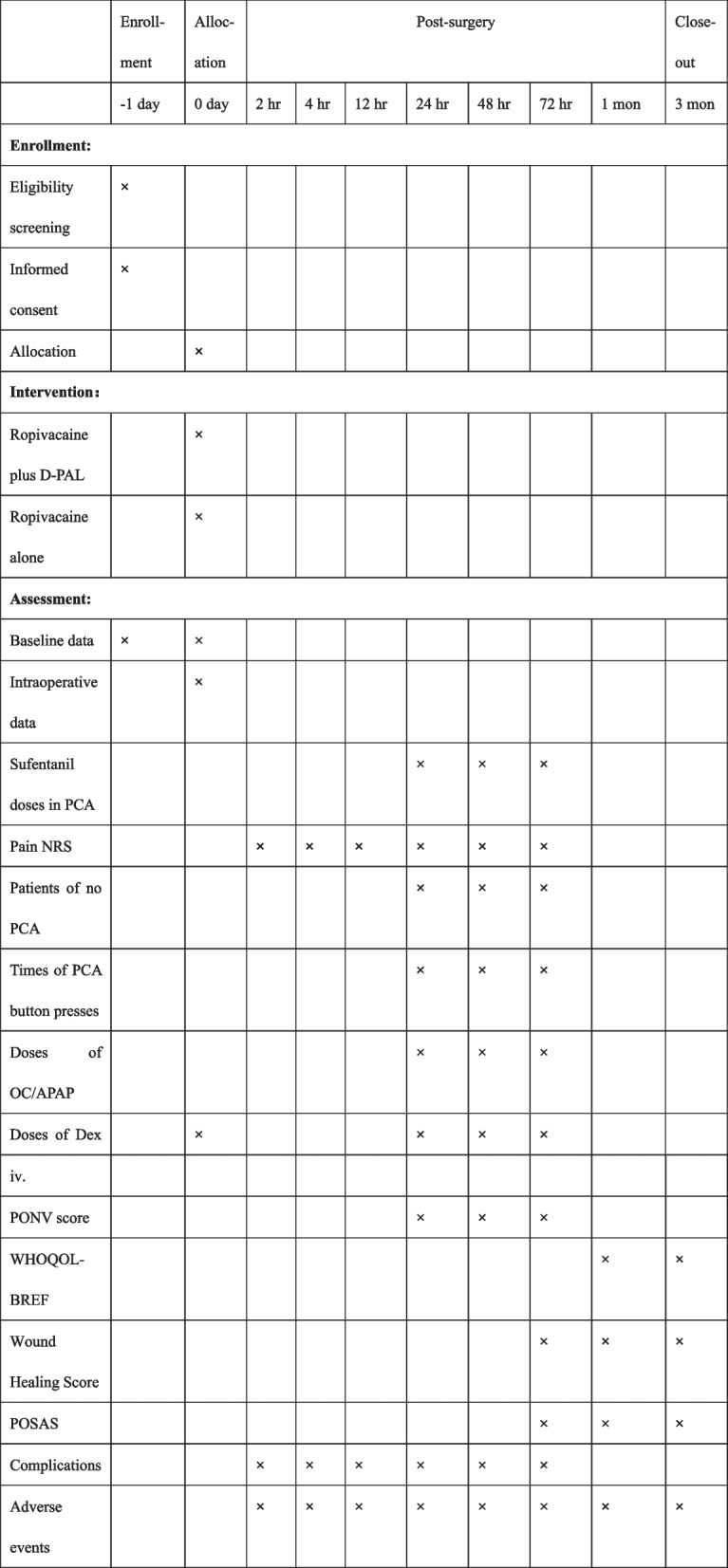
The schedule of enrollment, allocation and assessments. Abbreviations: D-PAL, dexamethasone palmitate emulsion; PCA, patient-controlled analgesia; NRS, numerical rating scale; OC/APAP, oxycodone /acetaminophen; Dex, dexamethasone; PONV, postoperative nausea and vomiting; WHOQOL-BREF, World Health Organization QoL abbreviated version; POSAS, Patient and Observer Scar Assessment Scale; hr, hour; mon, month; iv, intravenous

**Fig. 2:**
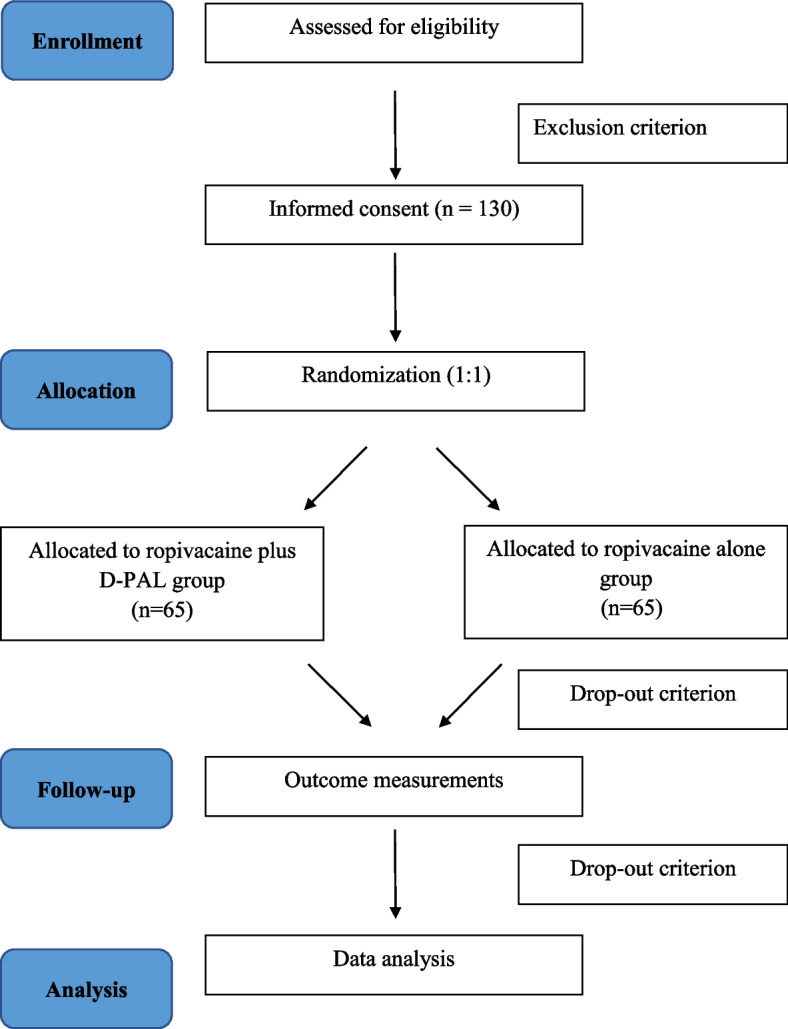
CONSORT flow diagram of the trial

This trial is a prospective, single-center, randomized, open-label, and blinded-endpoint study. Participants will be assigned to either ropivacaine plus D-PAL group or the ropivacaine alone group in an allocation ratio of 1:1 for this superiority trial. The trial will be conducted at Beijing Tiantan Hospital, Capital Medical University, China, from October 2022. We have used the SPIRIT reporting guidelines [30] and completed SPIRIT checklist (Supplementary file 1) and SPIRIT figure (Fig. 1). The flow diagram is illustrated in Fig. 2.

### Objectives

The primary objective of this trial is to determine whether preemptive incisional infiltration with ropivacaine plus D-PAL is superior to ropivacaine alone in relieving postoperative pain for adults undergoing neurosurgical craniotomy. The effects of both interventions on postoperative pain management and patients’ safety and quality of recovery will also be compared.

### Recruitment and ethics

The study plan is in accordance with “Declaration of Helsinki” and approved by the Ethics Committee of Beijing Tiantan Hospital (KY-2018-034-02-8). The strategy has been registered at ClinicalTrials.Gov (NCT04488315, Principal investigator: Fang Luo, Date of registration: July 19, 2020). This trial does not involve biological specimens, and protocol modifications will be not expected. All patients scheduled for elective supratentorial craniotomy for resection of a tumor, clipping of an unruptured aneurysm, or removal of an epileptic focus will be recruited and screened for participation by one researcher. This independent research will also be responsible for obtaining the informed consent by visiting the eligible patients 1 day before surgery and providing them with a verbal explanation of the written consent. Each participant will have sufficient time to ask any questions or concerns regarding this study and then decide whether to participate in this study. Eligible patients will sign informed consent and participants will have the right to withdraw their consent or discontinue participation without restrictions at any time point throughout the study. The confidentiality of participant data will be protected and patients will have the right to obtain relevant information and decide whether their data is to be shared with the regulatory authorities on reasonable request.

### Eligibility criteria

#### Inclusion criteria

- Scheduled for elective supratentorial craniotomy with an anticipated duration of > 4 h
- American Society of Anesthesiologists (ASA) physical status of I-–II
- Age 18-64 years
- Anticipated tracheal extubation, full recovery, and cooperation within 2 h postoperatively

#### Exclusion criteria

- Preoperative Glasgow Coma Scale < 15
- History of craniotomy
- Unable to comprehend the pain Numerical Rating Scale (NRS)
- Extreme body mass index (BMI) of < 15 or > 35
- Peri-incisional infection
- Allergy to dexamethasone, ropivacaine, or other analgesics
- History of alcohol or drugs abuse (more than 2 weeks)
- History of psychiatric disorders, uncontrolled epilepsy, or chronic headache
- History of severe cardiopulmonary, renal, or liver dysfunction
- History of radiation therapy and chemotherapy or with a high probability of such treatment postoperatively
- Pregnant or breastfeeding
- Refusal to give written informed consent

#### Withdrawal criteria

- Operation duration ≤ 4 h
- Not awake or extubated within 2 h after surgery
- Unexpected radiation therapy or chemotherapy postoperatively
- Revision for hematoma or brain swelling except wound problems within 72 h after surgery
- GCS < 15 within 72 h after surgery
- Poor cognitive function within 72 h after surgery
- Having a fever (≥ 39°C) within 72 h after surgery
- Lost in follow-up
- Voluntary withdrawal

### Randomization and blinding

Randomization sequence will be generated by a designated staff not involved in outcome data collection and analysis, who will use the block randomization method via SPSS version 22.0 (International Business Machines Inc., USA), with an electronic table of random numbers to allocate eligible participants to either of the two groups (in a 1:1 ratio). The allocation sequences will be prepared and be kept inside sealed, opaque, and consecutively numbered envelopes. The envelope will be opened by the neurosurgeons in charge, who will then prepare the study solution based on the respective allocation scheme and be therefore made aware of group assignations. Participants will be enrolled by a dedicated research nurse not involved in the data collection and analysis, and the neurosurgeons in charge are responsible for assigning participants to interventions. This is an open-label design with only patients and independent researchers in charge of follow-up and data analysis blinded to allocation. The D-PAL is a lipid emulsion in the infiltration solution, which can be easily distinguished from the clear ropivacaine alone study solution. Therefore, unblinding will not occur.

Prior to the commencement of the study, all investigators will receive standardized training on trial content, treatment strategies, evaluation, and quality control. All interventions will be carried out in accordance with clinical practice guidelines. During preoperative visit, eligible patients should be acquainted with pain NRS ranging from 0 (no pain) to 10 (worst possible pain) [31]. They will be guided on how to use a patient controlled analgesia (PCA) device.

### Intervention description

The neurosurgeons in charge will be responsible for the preparation of the respective drugs in a sterile fashion: 2 mL D-PAL emulsion (4.0 mg as D-PAL/mL is equivalent to 2.5 mg as dexamethasone; by Mitsubishi Tanabe Pharma Korea Co., Ltd) and 15 mL of 1% ropivacaine (Nai Le Pin ® 10mg/mL; by AstraZeneca AB, Sweden) diluted to a total volume of 30 mL in normal saline for ropivacaine plus D-PAL group, and 15 mL of 1% ropivacaine diluted to a total volume of 30 mL in normal saline for ropivacaine alone group.

### Anesthesia management

All patients will follow a standard anesthesia technique. In the operating room, standard monitoring will be continuously established, which includes electrocardiography, pressure (BP), heart rate (HR), pulse oxygen saturation (SpO_2_), bispectral index (BIS), and temperature. All patients will be preoxygenated with FiO_2_ > 0.8 before anesthesia induction with intravenous midazolam 0.05 mg/kg, sufentanil 0.3-0.5 μg/kg, propofol 1.5-2 mg/kg, and cisatracurium 0.2 mg/kg. After endotracheal intubation, anesthesia will be maintained with sevoflurane (0.4 MAC) and propofol (2-4 mg/kg∙h) to keep BIS values 40-60. The ventilation protocol consists of volume-controlled mechanical ventilation (Datex Ohmeda S/5 Advance, General Electric Healthcare, Helsinki, Finland) with an air-oxygen mixture (60:40), TV of 6-8 mL/kg, inspiratory to expiratory ratio of 1:2, fresh gas of 1-2L/min and a respiratory rate adjusted to normocapnia (PaCO_2_ between 35 and 45 mmHg). Intraoperative analgesia will be provided with continuous remifentanil infusion (0.1-0.3 μg/kg∙min) until the removal of Mayfield head holder at the end of surgery. Remifentanil infusion dose will be adjusted to attenuate potent stress responses and to keep mean arterial pressure (MAP) and HR within ± 20% of baseline values. Tramadol 100 mg will be administered intravenously before skin closure to prevent postoperative shivering and remifentanil-induced hyperalgesia, and ondansetron 4 mg will be given simultaneously to prevent tramadol-induced nausea and vomiting. No additional analgesics and antiemetics will be administered intraoperatively. Intraoperative hypertension and/or tachycardia will be treated with esmolol 0.5 mg/kg or nicardipine (10 μg/kg). Bradycardia (< 50/min) will be treated with atropine bolus 0.01 mg/kg. Muscle relaxants will be used as needed intraoperatively and the residual neuromuscular block will be antagonized with neostigmine 0.04 mg/kg and atropine 0.02 mg/kg after spontaneous ventilation recovery postoperatively. Patients will be shifted to the Post Anesthesia Care Unit (PACU) for monitoring after successful extubation, with stable hemodynamic, respiratory, and neurologic conditions.

### Pre-incisional infiltration

Patients will receive pre-emptive infiltration by the neurosurgeon immediately before head fixation in a sterile fashion. A 22-gauge needle will be introduced into the planned incision site, as well as each pin fixation site of the Mayfield head holder, at a 45° angle and throughout the entire thickness of the scalp. The ropivacaine plus D-PAL group will receive 0.027% D-PAL and 0.5% ropivacaine, whereas the ropivacaine alone group will be injected with 0.5% ropivacaine alone. The total volume of the local infiltrated solution will be at the discretion of the neurosurgeon, based on the length of the incision.

### Additional interventions

Patients will be evaluated to check fitness for NRS assessment 1 h after shifting to PACU, using a modified questionnaire of the Short Orientation Memory Concentration (SOMC) test [32]. The cognitive function of patients will be rated as good, able to recall and count with minimal mistakes (1-3); fair, with ≥ 3 mistakes; and poor, not able to recall at all. For patients regarded as poor, the same test will be repeated 1 h later. If the grading is poor again, then that patient will be withdrawn from the study [33]. A  PCA device containing sufentanil 200 μg and ondansetron 16 mg in 100 mL saline will be set up to deliver 1 mL as an intravenous bolus with a 10-min lockout interval after craniotomy. The maximum dose will be limited to 8 μg per hour, and there will be no initial dose or background infusion. Patients will be advised to push the analgesic demand button if they feel pain and to repeat it until the pain is relieved. PCA regimen will be discontinued when it is no longer needed. Each press will be recorded by an electronic memory system, including both valid and invalid presses. Patients will be given an oral supplementary tablet of oxycodone (OC)/acetaminophen (APAP) 5/325 mg (Mallinckrodt Inc., USA) for rescue analgesia when NRS score > 4 after receiving four times of bolus with the PCA device. OC/APAP will be prescribed at an interval of at least 6 h until the end of our study. Total doses of rescue analgesia consumed will be recorded.

### Follow-up

Follow-up will last 3 months postoperatively, during which an independent researcher blinded to the patient’s group allocation will conduct the following evaluations at different time points: pain NRS, sufentanil consumption in the PCA device, time to first PCA demand button press, cumulative consumption of OC/APAP, PONV scores, wound healing scores, World Health Organization QoL abbreviated version (WHOQOL-BREF), Patient and Observer Scar Assessment Scale (POSAS) scores, etc [34]. Adverse effects related to the surgery, anesthesia, steroid, or analgesics will be monitored and documented. Follow-up will be performed by telephonic communication after discharge, at 1 and 3 months postoperatively.

### Outcome measures

#### Baseline data

The demographic characteristics include age, gender, BMI, ASA status, neurological pathology, and comorbidity. Surgery and anesthesia characteristics include surgical site, length of incision, volume of local infiltration solution, duration of surgery and anesthesia, intraoperative analgesics (sufentanil and remifentanil) consumption, perioperative hemodynamic parameters, cumulative intravenous dexamethasone prescribed by a neurosurgeon, etc.

#### Primary outcome

Primary outcome will be the pain NRS scores at 24 h after craniotomy.

#### Secondary outcome

Pain NRS scores at 2, 4, 12, 48, and 72 h after craniotomy;

Prolonged post-craniotomy pain NRS at 1 and 3 months;

Number of patients needing no sufentanil at 24, 48, and 72 h after craniotomy;

Total consumption of sufentanil with PCA device at 24, 48, and 72 h postoperatively;

Total number of times that patients press the PCA button, including valid and invalid presses at 24, 48, and 72 h postoperatively;

Time to first PCA button press after surgery;

Time to first rescue analgesia with OC/APAP after surgery;

Duration of hospitalization postoperatively.

PONV scores at 24, 48, and 72 h after surgery. PONV is rated by patients as 0, absent; 1, nausea not requiring treatment; 2, nausea requiring treatment; and 3, vomiting.

WHOQOL-BREF scores at 1 and 3 months after craniotomy. The WHOQOL-BREF is a questionnaire of 26 items, including social relationships (3 items), physical health (7 items), psychological health (6 items), environment (8 items), overall QoL (1 item), and general health (1 item). Each domain’s mean score can range between 4 and 20 with a higher score indicating a better QoL.

Local soft-tissue atrophy at 72 h, 1 and 3 months after craniotomy. It is presented as superficial atrophy of skin and adipose tissue, central discoloration of the skin, yellow subcutaneous deposits, small sebaceous glands, cessation of hair growth, or mauve colored periphery at the injection site.

Wound healing score at 72 h, 1 and 3 months postoperatively. Wound Healing Score is rated by an independent researcher as excellent, good, and suboptimal.

Patient and Observer Scar Assessment Scale (POSAS) at 72 h, 1 and 3 months after craniotomy [35]. POSAS consists of 2 numerical numeric scales, the observer component, and the patient component.

Other complications such as respiratory depression, allergic reaction, local or systemic toxicity, wound infection, hematoma, and injection site infection throughout the treatment and follow-up period.

### Sample size

Earlier studies have reported that incision-site infiltration with ropivacaine plus dexamethasone could reduce about 30-50% of postoperative pain severity compared with ropivacaine alone [6, 19, 22]. Based on these literatures and our clinical experience, we hypothesize that the pain NRS scores was approximately 2.0 ± 1.5 (mean ± standard deviation) scores in the ropivacaine alone group and attempt to detect a difference of 40% in pain NRS scores between groups at 24 h after craniotomy. Therefore, the pain scores would be about 1.2 ± 0.9 scores in D-PAL plus ropivacaine group at 24 h after craniotomy. PASS V.11 software (NCSS, Kaysville, UT, USA) is used for sample size calculation with *α* = 0.05, *β* = 0.1, and power = 90%. Considering a dropout rate of 20%, a total of 130 patients will be required in this trial (*n* = 65 per group).

### Safety assessment

In this study, adverse events (AEs), defined as negative or unintended clinical manifestations throughout the treatment and follow-up period, will be monitored and recorded in detail on a case report form (CRF). All AEs related to the surgery, anesthesia, steroid, or analgesics, together with the soft-tissue atrophy and local infection, which might be induced by scalp infiltration with the mixture of ropivacaine and D-PAL emulsion, should be monitored during the 3 months’ follow-up. The soft-tissue atrophy, clinically manifested as superficial atrophy of skin and adipose tissue, central discoloration of the skin, yellow subcutaneous deposits, small sebaceous glands, cessation of hair growth, or mauve-colored periphery at the site of injection [36], will be monitored by careful observation and will be evaluated partly by the Patient and Observer Scar Assessment Scale (POSAS) and Wound healing score at 72 h, 1 m, and 3 months after craniotomy. AEs will be treated and reported to the Institutional Review Board (IRB) as soon as possible. Study interventions-related AEs will be treated for free. The trial will be terminated immediately in case of serious life-threatening AEs leading to prolonged hospital stay or death.

## Data collection and management

Data collection and management will be performed via the hospitalized medical record system or CRF by an independent researcher. All data will be kept strictly confidential for research purposes only. Only the primary investigator can obtain final test data. Follow-up will be conducted on days 1, 2, and 3 and at months 1 and 3 by an independent and experienced researcher, either in person or by contact via telephone (after discharge). Participants who discontinue or deviate from intervention protocols will not be replaced. They will be allowed to withdraw their consent without any restriction at any time. Their data will be retained, including all the primary and secondary outcomes until termination, except for the cases that are unable to complete the assessment of the primary outcome at 24 h postoperatively.

All personal information and data about the participants will be recorded into the CRFs, which are coded by an identification number and stored in a secure cabinet throughout the trial to guarantee confidentiality. All data will be entered into the Excel form for analysis by two independent researchers with double-checking. The electronic data will be stored on a double password-protected computer. Only the primary investigator and the statistician will have access to the files.

## Data Monitoring Committee (DMC)

This trial will be monitored by an independent Data Monitoring Committee (DMC) composed of specialists in ethics, statistics, and methodology through regular interviews. The DMC will audit the assessment and collection of all data after 30%, 60%, and 100% of patient inclusions. DMC will have access to interim results. Any AE will be reported to both the DMC and the IRB for judgement. Although there are no anticipated problems that may be detrimental to the participants, serious life-threatening AEs leading to prolonged hospital stay or death will be reported and the study will be terminated immediately.

### Missing data

Multiple imputations will be used to handle missing data during the whole study.

## Dissemination plans

The trial results will be made public through publication in a scientific journal.

## Statistics

All statistical analyses will be performed by a statistician who is blinded to the entire study. All analyses will include the patients completing 3 months of follow-up and will follow the intention-to-treat principle. Data analysis will be performed using SPSS 26.0 (SPSS Inc., Chicago, IL, USA). Kolmogorov-Smirnov test will be used to check for normal distribution. Normally distributed continuous variables will be described as mean ± SD and abnormally distributed variables as median (interquartile ranges, IQRs). Categorical variables will be reported as the number or proportion of patients. The primary outcome will be the pain NRS scores at 24 h postoperatively. These continuous data will be compared by independent two-tailed *t* tests if they are normally distributed or by Mann-Whitney *U* test if they are abnormally distributed. Similarly, secondary outcomes including pain NRS scores at 2, 4, 12, 48, and 72 h after craniotomy, sufentanil consumption with PCA device, PONV scores, WHOQOL-BREF, wound healing score, and POSAS scores at different time points and the duration of hospitalization will be compared by independent two-tailed *t* tests as normally distributed data or by Mann-Whitney *U* test as skewed data. In addition, the time to first button press or to first requirement of OC/PAPA will be compared by log-rank test and reported as hazard ratios (HRs) with 95% CI in Kaplan-Meier curves. Safety analyses will be compared with the incidence of AEs recorded in the safety data set using Chi-square test or Fisher’s exact test. The same statistical methods will be used for demographic characteristics and baseline information. We will judge a *P* value of less than 0.05 as significant for all tests.

Two interim analyses will be conducted for assessment of efficacy and safety after completion of the first 40 and 80 patients. The efficacy of the primary outcome and the incidence of AEs will be compared between two groups, and the study discontinuation threshold will be set at *P* < 0.01 using the alpha-sparing technique (O’Brien-Fleming) for benefit or harm.

## Discussion

In this prospective, randomized controlled trial, we seek to determine whether pre-emptive scalp infiltration with the addition of D-PAL emulsion to ropivacaine would offer superior analgesic efficacy for post-craniotomy pain compared to ropivacaine alone. To the best of our knowledge, this is the first study to evaluate the effects of D-PAL emulsion in scalp infiltration for craniotomy. D-PAL emulsion, dexamethasone palmitate incorporated into lipid microspheres, has a stronger anti-inflammatory activity, as well as a longer duration of action and fewer side effects than free dexamethasone. The clinical utility of D-PAL emulsion has been reported to treat inflammatory diseases or pain diseases by intravenous administration or local infiltration [24, 25, 28, 29, 37]. In this study, we intend to choose the concentration of about 0.027% D-PAL (equivalent to 0.017% dexamethasone) for scalp infiltration, which seems to be the lowest possible concentration of dexamethasone for local use according to previous literatures [6, 37–39]; thus, it should be considered safe and help reduce the potential risks of local glucocorticoid, such as delayed wound healing and wound infection. It is true that systemic dexamethasone has become the standard pharmacological agent in the treatment of cerebral oedema associated with intracranial tumors [40]. Perioperative high-dose of dexamethasone in craniotomy has been reported to be associated with hyperglycemia, infection, peptic ulcer, and decreased incidence of PONV and pain [41]. All these may confound our findings. So the consumption of intravenous dexamethasone prescribed by the neurosurgeon should be calculated in the two groups.

Ropivacaine is the most widely used long-acting LAs for incisional infiltration [42]. In this study, we choose a widely used concentration of 0.5% ropivacaine for scalp infiltration in craniotomy [43, 44]. There are two main reasons for this. First, it is reported that scalp block using 0.5% ropivacaine has obtained preferable and prolonged postoperative analgesia compared to lower concentrations in craniotomy [45]. Second, ropivacaine is considered one of the safest LAs attributed to its lipophilicity, which leads to decreased toxicity in both cardiovascular and central nervous systems [46]. Moreover, the intradermal injection of 0.25-0.75% ropivacaine can induce peripheral vasoconstriction and decreases local blood flows at the injection [47]. It is reported that the peak plasm concentration of 0.5% ropivacaine occurs within 13 min after commencement of scalp infiltration without any signs of local anesthetic toxicity [40].

Mixtures of local anesthetics and steroids have been used in common practice. However, the safety of this mixture has not been considered carefully. Combination of non-particulate steroids with local anesthetics could form crystals in solution, which may be caused by alkalinization of steroids. The deposits of insoluble crystals could partly account for the depressed and atrophic skin at the site of injection [48]. However, it is recently reported that the mixture of D-PAL emulsion (with an almost neutral pH of 7.1) with ropivacaine produced no significant particulates under fluorescence microscopy [20], suggesting an optimal and safe combination for use in our protocol. As to the aseptic soft-tissue damage or infection after local injection, the occurrence and duration seem to be a function of local drug concentration [49]. In this study, we intend to choose the lowest possible concentration of dexamethasone for local use to achieve a safe regimen. To our knowledge, no serious local AEs have been reported concerning local infiltration with the mixture of D-PAL and ropivacaine [50]. However, the actual number of trials is limited, and therefore, additional studies regarding its potential safety and efficacy are needed in the future.

Craniotomy site may be a determinant for the incidence and intensity of post-craniotomy pain, which is partly explained by the anatomical location of peri-cranial muscles being damaged. Studies suggest that patients undergoing infratentorial procedures may have more pain than those submitted to a supratentorial approach [2]. Further studies indicate that supratentorial craniotomy involving temporal muscle incision is assumed to be more painful, while the procedure involving frontal muscle incision tends to induce the lowest pain [51]. Therefore, we specify the accurate location in this trial and restrict the surgical approach to be supratentorial.

There are some limitations in our study. Firstly, this is a single-center study. However, ours is a large institution in Asia that specializes in neurosurgery and offers clinical, scientific, and teaching base of neurosurgery, which represents a great advantage in generalizability of our outcomes in this field. Secondly, neurosurgical patients have high levels of anxiety and depression, which are also possible risk factors for post-craniotomy pain [52]. However, due to the absence of a specialized assessor preoperatively, we will not able to control for these factors. Thirdly, we only select a single concentration of D-PAL as an infiltration adjuvant in this study. Future trials on dose-dependent effects of D-PAL should be warranted to determine the optimal dose. Fourthly, D-PAL is a non-transparent emulsion, and thus, surgeons and anesthesiologists will not be blinded in this study. Future studies should attempt to prepare the infiltration solution in non-transparent syringes. Fifthly, the sample size is not big enough to identify the risks of rare but important side effects, such as allergic reactions, systemic toxicity, infection, peptic ulcer, and changes in wound healing. A systemic review is the only way to establish these risks. Finally, an overview of case reports has determined that, in about half of cases with tissue atrophy or infection after local corticosteroid injection, the affected area returns to normal over a few months to years [53]. In this study, follow-up evaluations will be conducted within 3 months postoperatively, on account of the fact that we have selected a low concentration of dexamethasone for scalp infiltration, and the safety of local injection with dexamethasone and Diprospan has already been demonstrated in our previous work [6, 19]. However, it is suggested that the follow-up visit should be extended beyond 3 months in case of tissue atrophy or infection after local injection.

In conclusion, post-craniotomy pain is a common occurrence associated with poor outcomes. At present, there is no generally accepted opinion regarding the most suitable analgesic method. If our results reveal a significant decrease in postoperative analgesics with the addition of D-PAL emulsion in pre-incision infiltration, this trial will provide an effective alternative to optimize pain outcome for patients undergoing craniotomy.

## Trial status

Not recruiting. Protocol (V3.2/2018-07-05). The recruitment will be expected to begin on October 1, 2022, and to be completed on May 1, 2023.

## Supplementary Information

**Additional file 1.** SPIRIT checklist.

## Data Availability

The datasets which will be used and/or analyzed during this study are available from the corresponding author on reasonable request.

## Abbreviations

D-PAL: Dexamethasone palmitate emulsion
PCA: Patient-controlled analgesia
NRS: Numerical rating scale
OC/APAP: Oxycodone /acetaminophen
Dex: Dexamethasone
PONV: Postoperative nausea and vomiting
WHOQOL-BREF: World Health Organization QoL abbreviated version
POSAS: Patient and Observer Scar Assessment Scale
BP: Blood pressure
HR: Heart rate
SpO_2_: Pulse oxygen saturation
BIS: Bispectral index
NSAIDs: Nonsteroidal anti-inflammatory drugs
